# The Transcriptional Response of Neurotrophins and Their Tyrosine Kinase Receptors in Lumbar Sensorimotor Circuits to Spinal Cord Contusion is Affected by Injury Severity and Survival Time

**DOI:** 10.3389/fphys.2012.00478

**Published:** 2013-01-09

**Authors:** M. Tyler Hougland, Benjamin J. Harrison, David S. K. Magnuson, Eric C. Rouchka, Jeffrey C. Petruska

**Affiliations:** ^1^Department of Anatomical Sciences and Neurobiology, University of LouisvilleLouisville, KY, USA; ^2^Laboratory of Neural Physiology and Plasticity, Kentucky Spinal Cord Injury Research Center, Department of Neurological SurgeryLouisville, KY, USA; ^3^Laboratory of Locomotor Systems and Rehabilitation, Kentucky Spinal Cord Injury Research Center, Department of Neurological SurgeryLouisville, KY, USA; ^4^Department of Computer Engineering and Computer Science, University of LouisvilleLouisville, KY, USA; ^5^Laboratory of Bioinformatics, Department of Computer Engineering and Computer ScienceLouisville, KY, USA

**Keywords:** spinal cord injury, neurotrophins, neurotrophin receptors, contusions, transcription, injury mechanisms, sensory neurons, genetic regulation

## Abstract

Traumatic spinal cord injury (SCI) results in changes to the anatomical, neurochemical, and physiological properties of cells in the central and peripheral nervous system. Neurotrophins, acting by binding to their cognate Trk receptors on target cell membranes, contribute to modulation of anatomical, neurochemical, and physiological properties of neurons in sensorimotor circuits in both the intact and injured spinal cord. Neurotrophin signaling is associated with many post-SCI changes including maladaptive plasticity leading to pain and autonomic dysreflexia, but also therapeutic approaches such as training-induced locomotor improvement. Here we characterize expression of mRNA for neurotrophins and Trk receptors in lumbar dorsal root ganglia (DRG) and spinal cord after two different severities of mid-thoracic injury and at 6 and 12 weeks post-SCI. There was complex regulation that differed with tissue, injury severity, and survival time, including reversals of regulation between 6 and 12 weeks, and the data suggest that natural regulation of neurotrophins in the spinal cord may continue for months after birth. Our assessments determined that a coordination of gene expression emerged at the 12-week post-SCI time point and bioinformatic analyses address possible mechanisms. These data can inform studies meant to determine the role of the neurotrophin signaling system in post-SCI function and plasticity, and studies using this signaling system as a therapeutic approach.

## Introduction

Traumatic injury to the spinal cord (SC) results in a variety of changes to sensorimotor circuits. Sensory neurons of the dorsal root ganglia (DRG) rapidly undergo long-lasting changes in their electrophysiological properties and growth capacity (e.g., Bedi et al., 2010, 2012; Walters, 2012). Locomotor circuitry in the SC caudal to an injury site undergoes plasticity at the cellular, synaptic, and connectivity levels in an activity-dependent manner after injury in humans and experimental models (e.g., Edgerton et al., 2004; Rossignol, 2006; Petruska et al., 2007). One strategy to restore function after spinal cord injury (SCI) is physical therapy and/or locomotor rehabilitation training (e.g., Wernig et al., 1995). The neurotrophins Nerve Growth Factor (NGF), Brain Derived Neurotrophic Factor (BDNF), and Neurotrophin 3 (NT3) are secreted growth factors that were first characterized for their important role in the survival of subpopulations of sensory neurons and in formation of SC sensorimotor circuits during development (e.g., Barbacid, 1995; Lindsay, 1996; Huang and Reichardt, 2001). In addition to these essential roles in establishing the physiological patterns of developing neural circuitry, neurotrophins are implicated as having a role in activity-dependent changes associated with restoration of function after SCI (described below).

Neurotrophins have key roles in modulating the anatomical, neurochemical, and physiological properties of cells in the central and peripheral nervous system. The effects of neurotrophins on responses to stimuli in both the intact and injured nervous system have been extensively investigated and studies have demonstrated an important role in modulation of sensorimotor physiology (for reviews, see Huang and Reichardt, 2001, 2003; Reichardt, 2006; Skaper, 2008, 2012). The neurotrophins have therefore become a frequent target for manipulation after injury. Delivery of exogenous BDNF and NT3 to the transected SC improves recovery of hindlimb function (Blits et al., 2003; in rats) and results in a level of function similar to that seen in animals receiving locomotor training after spinal transection (Boyce et al., 2007; in cats). Such demonstrations of enhanced post-SCI function in response to exogenous neurotrophins suggests a role for neurotrophin signaling in models of activity-dependent plasticity after injury, possibly including physical therapy. For example, in the lumbar SC of rats, post-SCI locomotor training causes an increase in both BDNF and NT3 above levels of non-trained animals (Hutchinson et al., 2004; Côté et al., 2011). In light of the demonstrated and suggested roles in modulating sensorimotor physiology, characterizing the endogenous regulation of neurotrophins and their receptors after injury is particularly relevant.

Neurotrophins influence cellular processes by binding to membrane-bound receptors which transduce the extracellular signal into intracellular effect – their high affinity tyrosine kinase receptors. In general, NGF binds TrkA, BDNF bindsTrkB, and NT3 binds TrkC (e.g., Barbacid, 1995; Patapoutian and Reichardt, 2001; Huang and Reichardt, 2003), although cross-talk is recognized and there is a low-affinity receptor, p75, which we do not consider here. To determine the role of neurotrophins in any process or condition one must examine not only the neurotrophins, but also the receptors.

Prior characterizations of changes in neurotrophins and Trk receptors in lumbar neural circuitry have been instrumental in elucidating the complex regulation of these important molecules after injury (Table 1). However, these have largely focused on time points of less than 6 weeks (Hayashi et al., 2000; Liebl et al., 2001; Nakamura and Bregman, 2001; Widenfalk et al., 2001; Qiao and Vizzard, 2002, 2005; Gulino et al., 2004; Zvarova et al., 2004; Qin et al., 2006; Li et al., 2007; Hajebrahimi et al., 2008; Qian et al., 2011; Keeler et al., 2012). Although valuable for elucidating the role of neurotrophin signaling in the first 6 weeks after SCI, these data are of uncertain value for relating to longer-term post-SCI function. Given the many demonstrations of continued changing conditions after SCI (e.g., Beattie et al., 2002; Profyris et al., 2004; Ung et al., 2008; Beck et al., 2010), it is important to recognize that the temporal character of experiments has a significant influence on the outcome.

**Table 1:** **Summary of recent experiments assessing expression levels of neurotrophins and neurotrophin receptors after SCI**.

PMID	Reference	Molecule(s)	Injury model	Injury site	Sampling site	Experimental methods	Post injury time course	Findings	Notes
10757326	Hayashi et al. (2000)	NGF, BDNF, NT3, TrkA, TrkB, TrkC	Spinal cord crush (60 g, 1 s)	Under T10 vertebra	Five segments centered on epicenter	ISH	Six times; up to 3 days	Increase in BDNF and NT3, weaker increase for NGF; TrkA and TrkC not detected; TrkB detected in non-neurons and motoneurons, and increased in both with SCI	Functional status of animals was not assessed, but reference was given to Guth et al., 1994 (model shows mild motor deficit after awakening from anesthesia with apparent full recovery at 72 h); BDNF observed in non-neurons after SCI; qualitative data only, no statistics
						qPCR (NGF only)		NGF increased weakly	

11161589	Liebl et al. (2001)	TrkA, TrkB, TrkC	12.5 g cm NYU contusion	Under T9, T10 vertebra	Entire SC	ISH	1 day	No difference in TrkA, TrkB, or TrkC expression rostral or caudal to injury	Absent Trk expression around injury site and reduced in penumbra, no statistics

11331375	Widenfalk et al. (2001)	NGF, BDNF, NT3, TrkA, TrkB, TrkC	25 g cm NYU contusion transection	Under T9 vertebra	Cross-sections taken from regions throughout length of spinal cord injury epicenter and up to 1 cm caudal	ISH	Six times; up to 6 weeks	No change in TrkA, TrkB, TrkC; increase in NGF and BDNF up to 1 day, but no change vs. intact at 6 week; NT3 not detected in either intact or injured spinal cord	Functional status of animals with contusion was not assessed; reports on multiple injury types; statistical analysis uses optical density measures for ISH, and radioactivity for RPA
					RPA (NGF, BDNF, NT3)	6 weeks after contusion	No change in NGF and BDNF; NT3 not detected	
							1 day after transection	Increase in NGF and BDNF; NT3 not detected	

11358454	Nakamura and Bregman (2001)	NGF, BDNF, NT3, NT4	Lateral over hemisection	Under T6 vertebra	Entire SC	RPA	Five times; up to 2 weeks	Increase in NGF and BDNF up to 4 days, NT3 and NT4 not detected	Used whole SC mRNA, no assessment of injury or post-SCI function, expression data represented as % GAPDH

12115676	Qiao and Vizzard (2002)	TrkA, TrkB	Transection	Under T8-T10 vertebrae	L1-S1 DRG	IHC	5–6 weeks	Increase in # of TrkA and TrkB positive cells in L1, L6/S1, no change at L4/5	No assessment of post-SCI function, data expressed as # of Trk-IR positive cells

15193526	Gulino et al. (2004)	BDNF, NT4	Lateral hemisection	Under T9 vertebra	L4/5 SC	IHC	Four times; up to 2 weeks	Decrease in BDNF, NT4 starting at 30 min, lasting up to 2 weeks	Coronal sections; no assessment of post-SCI function; data expressed as relative optical density of IR positive cells in ipsilateral vs. contralateral hemisected cord

15236239	Zvarova et al. (2004)	NGF, BDNF	Transection	Under T7–T9 vertebrae	T7-S1	ELISA	<1, 6 weeks	Increase in NGF T7–T8 (rostral), and T13-L1, L6-S1 (caudal) 6 weeks post injury; Increase in NGF T9-T10 (caudal), and T13-L1, L6-S1 (caudal) < 1 week post injury; increase in BDNF T7-T10, T13-L1, L6-S1 6 weeks post injury; Increase in BDNF T7-L1, L3-S1 < 1 week post injury	No assessment of post-SCI function; neurotrophin concentration expressed as proportion of total protein

15611995	Qiao and Vizzard (2005)	TrkA, TrkB	Transection	Under T8-T10 vertebrae	L1-S1 DRG	IHC	2 days and 2 weeks	Increase in # of TrkA and TrkB positive cells in L1, L6/S1, no change at L4/5	No assessment of post-SCI function; data expressed as # of Trk-IR positive cells

17055159	Qin et al. (2006)	NGF, BDNF, NT3	Lateral hemisection	Under T10 vertebra	Ventral horn caudal to T10 injury site	IHC	Three times; up to 3 weeks	Increase in # of BDNF, NT3, NGF positive cells in ventral horn	Characterized injuries by spared function (BBB locomotor score); data expressed as optical density of IR positive cells in hemisected cords relative to control cords

17459471	Li et al. (2007)	NGF, BDNF, NT3	Transection	Under T9–T10 vertebra	Laminae I-IX, ∼1.5 cm caudal to injury site	IHC	Four times; up to 3 weeks	Increase in # of NGF IR cells and relative IR in laminae I-IX up to 3 weeks, # of NT3 IR cells and relative IR in laminae VIII and IX up to 3 weeks and laminae I–VII at 2 weeks, # of BDNF IR cells and relative IR in laminae I-IX up to 7 days	Characterized injuries by spared function (BBB locomotor score); data expressed as relative optical density of IR positive cells in hemisected vs. control cords

18585435	Hajebrahimi et al. (2008)	NGF, BDNF, NT3, TrkA, TrkB, TrkC	25 g cm NYU contusion	Under T9-T10 vertebra	∼1 cm block of SC	EthBr staining intensity	Eight times; up to 3 weeks	Decrease in NGF after 6 h that increases until 3 week where greater than control, BDNF and NT3 decrease after 6 h up to 3 weeks; TrkA, TrkB, TrkC decrease up to 3 weeks	B2m used as internal control; no assessment of injury or post-SCI function; data expressed as levels of mRNA relative to B2m

21441969	Qian et al. (2011)	TrkC	Transection/resection	Under T8–T9 vertebrae	Motor cortex; SC: adjacent to injury, rostral/caudal to injury	mRNA, protein	Four times; up to 2 weeks	Decrease at and around injury site from 1 to 7 days, then rapid increase until 14 days	mRNA more highly expressed at injury site than neighboring segments; protein shows same pattern; no assessment of post-SCI function

22244304	Keeler et al. (2012)	BDNF, NT4, NT3, TrkB, TrkC	Transection	Under T9 vertebra	L4–L6 SC (laser-captured motoneurons, select laminae); L4–6 DRG (large neurons)	Laser-capture, qPCR, WB	up to 31 days	Increase in NT4 and TrkB mRNA at 10 days, NT4 mRNA at 31 days; increase in NT3, NT4, BDNF protein at 31 days; increases in NT4 at 10 days, TrkB at 31 days in motoneurons; no change in expression in intermediate gray matter or large DRG neurons	More robust increase in expression after exercise; no assessment of post-SCI function; data expressed as mRNA or protein relative to control

	Hougland et al. (this article)	NGF, BDNF, NT3, TrkA, TrkB, TrkC	12.5 and 25 g cm NYU contusion	Under T9 vertebra	L4/5 spinal cord and DRG	qPCR	6 and 12 weeks	Gene regulation differed by injury severity and by post-SCI time; correlated expression of genes at 12 weeks in DRG	Characterized injuries by spared function (BBB locomotor score) and by histology (white matter sparing); examined co-regulation of genes

*Abbreviations: n.c, no change; IHC, immunohistochemistry; ISH, *in situ* hybridization; WB, western blot; RPA, ribonuclease protection assay; EthBr, ethidium bromide; LCM, laser-capture microdissection; qPCR, quantitative polymerase chain reaction; SC, spinal cord; DRG, dorsal root ganglion*.

The impact of SCI also varies depending on the location of the injury itself and the spatial relation of the investigated tissue to the SCI. Clearly, the relative composition of types of tissues innervated changes throughout the course of the neuraxis as does the specific function of local circuitry. For example, in rat, the spinal components of bladder control are focused on the T13/L1 and L6/S1 segments, colon function is focused in L6/S1, and the locomotor central pattern generator appears focused in (though not limited to) the L1/2 segments, spinal sympathetic circuitry regulating outflow exists roughly from T1-L2, and spinal parasympathetic circuitry exists in the sacral-caudal SC. Thus it follows that the effect on spared function and/or recovery is influenced by the level of the injury (e.g., Magnuson et al., 1999, 2005; Garcia-Alias et al., 2006), but this also extends to less direct functions (Campagnolo et al., 2000; Lucin et al., 2007). It is also very important to consider that both neural and non-neural tissues remote from the SCI can be affected (e.g., Collazos-Castro et al., 2005; Massey et al., 2006; Gris et al., 2008).

Sensory input to the SC plays a role in establishing natural and therapy-induced recovery and regulating spinal function in the absence of descending control. For example, urinary bladder function after SCI is highly reliant on sensory input and plasticity of sensory afferents (e.g., Tai et al., 2006; de Groat and Yoshimura, 2009), and SCI affects the trk receptor profile of neurons in DRG segments innervating bladder differently than for DRG innervating hindlimb (Qiao and Vizzard, 2002, 2005), a finding that extends to spinal trk receptors as well (Zvarova et al., 2004). Additionally, the type and amount of sensory input can influence spontaneous recovery after SCI (e.g., Grau et al., 2004, 2012; Ollivier-Lanvin et al., 2010; Caudle et al., 2011; Ferguson et al., 2012a,b) and also influence the effectiveness of physical therapy (e.g., Bouyer and Rossignol, 1998, 2003; Edgerton et al., 2004, 2008; Gomez-Pinilla et al., 2004; Frigon and Rossignol, 2009; Ollivier-Lanvin et al., 2010), all of which may involve neurotrophin signaling (e.g., Gomez-Pinilla et al., 2004; Hutchinson et al., 2004; Boyce et al., 2007, 2012; de Leon, 2007; Côté et al., 2011). Further, autonomic dysreflexia (AD), a life-threatening condition that is common for those living long-term with cervical or high thoracic SCI, is triggered most frequently by nociceptive sensory input (Maiorov et al., 1998; Krassioukov and Fehlings, 1999; Garstang and Miller-Smith, 2007), and sprouting of central terminals of nociceptive neurons, purportedly modulated by NGF, is proposed as a mechanism contributing to AD (Weaver et al., 1997; Krenz et al., 1999; Marsh et al., 2002; Cameron et al., 2006; Ackery et al., 2007). It is important, therefore, to examine not only the SC, but also the sensory neurons providing information to the SC, and to consider that the effects of SCI on these neurons may differ with their spatial relation to the SCI, and/or to the different tissues they innervate (e.g., Qiao and Vizzard, 2002; Zvarova et al., 2004; Bedi et al., 2010, 2012; Keeler et al., 2012). The spatial character of experiments, in terms both of the level of SCI and the relation to the SCI of the tissue investigated, has a significant influence on the outcome.

Injury severity, or more precisely the degree and nature of the tissue spared after injury, is one of the key factors determining the functional capabilities of the SC caudal to the SCI. The literature is replete with examples of this when reports are considered together (e.g., Rossignol and Frigon, 2011). Far fewer single studies examine multiple injury severities (e.g., Magnuson et al., 2005; Smith et al., 2006), although the injury severity character of experiments has a significant influence on the outcome.

We sought to characterize the natural regulation of neurotrophin and trk receptor genes in tissues and conditions that were applicable to experimental studies of long-term function and recovery after SCI and to the human condition. We therefore characterized the transcriptional response of neurotrophins and their cognate Trk receptors to SC contusion temporally (6 and 12 weeks post injury), spatially (in lumbar SC and DRG), and relative to injury severity (12.5 and 25 g cm NYU contusions).

## Materials and Methods

All experimental protocols and procedures were approved by the Institutional Animal Care and Use Committee at the University of Louisville, Louisville, KY, USA. Experimental animals were 7 week old female Sprague–Dawley rats (Taconic Labs, Hudson, NY, USA). Animals were housed in pairs throughout the course of our experiments.

### Surgical spinal cord injury

Rats (*n* = 47) were anesthetized with 50 mg/kg sodium pentobarbital (Sigma, St Louis, MO, USA). Once sedated, Lacquer Lube was applied to the eyes to prevent drying. After skin incision, laminectomy was performed at vertebral level T9, to expose the T10 SC. Contusion injuries were produced using the New York University (NYU) Impactor. Either “Moderate” or “Moderately severe” injuries were produced by releasing a 10 g, 2 mm rod from 12.5 or 25 mm height, respectively, onto the exposed dura mater of the SC. These will subsequently be referred to as 12.5 and 25 g cm injuries. After producing the contusion the wound was closed in layers and the skin incision was stapled. Rats received fluids (10cc 0.9% saline subcutaneously), and antibiotic treatment (0.1cc Gentamicin (50 mg/mL) intramuscularly, and Bacitracin was topically applied on the incision site). Animals were housed overnight in a recovery room with a heating pad under their cage, and were taken to the animal facilities in the morning.

Assessment of mRNA expression in SCI animals was compared to control animals. These consisted of naïve rats (two per time point group) and rats receiving laminectomy-only (three rats per time point group), for a total of five controls per time point. There were four additional laminectomy-only control rats included with the animals used for the 6-week post-SCI DRG assessment. All surgical procedures (except for the SCI), were as described above for the laminectomy-only control rats.

### Injury characterization

#### Behavior

Experiments were performed on rats separated into groups based on injury severity, survival time, and the tissue to be analyzed for mRNA expression. Rats were familiarized with the testing procedures and personnel by handling for 1 week before injury. Pre-surgical behavioral assessments were done to ensure no pre-existing conditions were present that would subsequently affect our locomotor outcome measures. Seventeen rats received 12.5 g cm NYU (moderate) and 16 rats received 25 g cm NYU (moderately severe) injuries. Hindlimb locomotor function was assessed with the Basso, Beattie, and Bresnahan (BBB) Locomotor Rating Scale (Basso et al., 1996). BBB testing was carried out prior to injury and 7, 14, 21, 28, 35, 42 for the 6-week SC group, and 7, 14, 21, 28, 35, 42, 49, 56, 63, 72, 79, and 84 days post injury for the 12-week SC groups, 12 week DRG group, and at 7, 14, 28, and 42 days post injury for the 6-week DRG group. For testing, rats were placed in an open field (a plastic tank that was 105 cm in diameter with 30 cm high walls) for 4 min. BBB testing was done after animal care in the morning. Hindlimb movement and locomotion were scored simultaneously by two observers who were blind to the treatment groups. We include the BBB measures as a means to characterize the injuries with commonly used assessments so that the mRNA measures can be placed in context.

#### Histology

At the end of the testing period, rats were anesthetized with sodium pentobarbital and euthanized via transcardial perfusion with 30% RNA Later (Qiagen) in 0.1 M Phosphate Buffered Saline (PBS). An approximately 10 mm long block of SC containing the injury epicenter was removed from each animal and immersed in 4% paraformaldehyde. After 1 week cords were immersed in PBS containing 30% sucrose for cryoprotection until further processing. For sectioning, tissue was embedded in TissueTek^®^ (VWR) and frozen. The blocks were cut 50 μm thick in the transverse plane on a cryostat and were sampled every 250 μm. A series of sections spanning the rostrocaudal extent of the lesion was stained with eriochrome cyanine (EC) to assess amounts of spared myelin as described (Rabchevsky et al., 2007). Light microscopy was used to determine spared white matter (SWM). Images were captured using a SPOT digital camera (Diagnostic Instruments) mounted on a Zeiss Axioskop. From these, the area of spared tissue was manually designated (Intuos drawing tablet; Wacom, Otone, Japan). Areas of white matter sparing were calculated using the ImageJ program and expressed as a proportion of control (defined as group mean of the smallest white matter area from an analogous section of SC from all control animals). For each injured animal, the SCI epicenter was defined quantitatively as the section containing the least amount of intact tissue. Percent white matter sparing is reported as mean (±SD). As with the BBB, we include the WMS measures as a means to characterize the injuries with commonly used assessments so that the mRNA measures can be placed in context.

### mRNA expression

#### Isolation and cDNA conversion

Animals were euthanized after final behavioral assessments and exsanguinated by transcardial perfusion using 30% RNA later (Qiagen) in PBS. Lumbar SCs (L4/5) and DRG were removed and immersed in 100% RNA later and stored at −20°C until further processing. SCs were homogenized on ice in 1 mL Trizol and RNA was isolated using Trizol/chloroform extraction method. Briefly, homogenate was transferred to a 1.5 mL tube and spun at 12,000 *g* for 10 min at 2°C. The supernatant was transferred to a new tube and 200 μL chloroform added. This mixture was spun for 15 min at 2°C to separate into aqueous and organic phases. The aqueous phase was transferred to a new tube and alcohol precipitation was performed with 100% isopropanol, then 70% ethanol. After removal and drying of excess ethanol, the pellet was resuspended in 30 μL nuclease free H_2_0, solubilized in 600 μL Buffer RLT with beta-Mercaptoethanol (BME), and processed through RNeasy MiniKit (Qiagen) per manufacturers protocol. DRGs were homogenized directly in Buffer RLT + BME and processed through RNeasy MiniKit. RNA was analyzed by Nanodrop (ThermoScientific, Waltham, MA, USA) to obtain concentration and 500 ng of RNA from each sample was reverse transcribed into cDNA using Quanta Biosciences qScript cDNA SuperMix. All RNA was converted to cDNA using the same lot of reverse transcriptase. Performing the reverse-transcription for all samples with the same reagents is a methodological procedure meant to reduce the cross-sample variability which in turn can enhance the reliability of statistical assessments.

#### qRT-PCR

mRNA expression levels were quantified by qRT-PCR on Corbett Research 6000 (Qiagen) using FastStart Universal SYBR Green Master Mix(Roche). Duplicate reactions were run for each sample for both the gene of interest and the normalizer [Beta-3 Tubulin – demonstrated as a suitable normalizer gene for SCI work (Strube et al., 2008)]. Relative expression levels were calculated as ΔΔCT of gene of interest vs. normalizer. Primer sequences for the genes analyzed are provided in Table 2, along with their relationship to the known gene structure and transcript species.

**Table 2:** **Primer sequences for qPCRand relationship to gene/transcripts**.

Gene	Forward primer	Location of forward primer	Reverse primer	Location of reverse primer	Gene intervals probed	Isoforms probed
TrkC	GATTCAGGGAACAGCAATGG	Within exon 1	TTGATGGTCAGCTTCTGGAG	Spans exons 2–3	Bp 231–382 of NM_001270656	All isoforms

NT3	CGATCTTACAGGTGAACAAGG	Spans exons 1–2	CTGGCAAACTCCTTTGATCC	Spans exons 2–3	Bp 177–318 of NM_031073	Full length NT3

TrkB	CATTGACCCAGAGAACATCAC	Within exon 2	TCGAGTGAAATTGATGTGCC	Spans exons 4–5	Bp 846-1032 of NM_012831.2	Full length TrkB

BDNF	CGAGACCAAGTGTAATCCCA	Within exon 2	TCTATCCTTATGAACCGCCA	Within exon 2	Bp 920–1075 of NM_001270630.1	All isoforms

TrkA	TTCAGTGATACCTGTGTCCAC	Spans exons 12–13	GACGAGCATTCTCAGATGTC	Spans exons 13–14	Bp 1558–1732 of NM_021589.1	All isoforms

NGF	CTTCAACAGGACTCACAGGA	Within exon 3	TTGTTAATGTTCACCTCGCC	Within exon 3	Bp 517-681 of XM_003749364.1	All isoforms

TubB3	CGAGTGAAGTCAGCATGAG	Within exon 1	ACATACTTGTGAGAGGAGGC	Spans exons 2–3	Bp 36–228 of NM_139254.2	All isoforms

### Statistics

Statistical analyses were performed using SPSS (IBM, North Castle, NY, USA) or SigmaPlot/SigmaStat (Systat Software, San Jose, CA, USA). A Student’s *t*-test was performed to determine if expression levels differed between control groups. In cases where gene expression did not differ between control groups the 6- and 12-week control groups were combined and the expression values for the experimental groups are reported as a fold-change of the unified control group. One-way analysis of variance (ANOVA) was performed on these values with *post hoc* Tukey’s test for all pairwise comparisons. All groups with *p* < 0.05 difference are reported as significant. Pearson Product Moment was calculated to determine the relationships between the expression levels of the different transcripts, and to determine the relationships between BBB/WMS vs. expression levels. Differences between BBB scores were assessed using a mixed model repeated measures ANOVA with a *post hoc* Bonferroni *t*-test.

## Results

### Injury characterization

**Figure 1:**
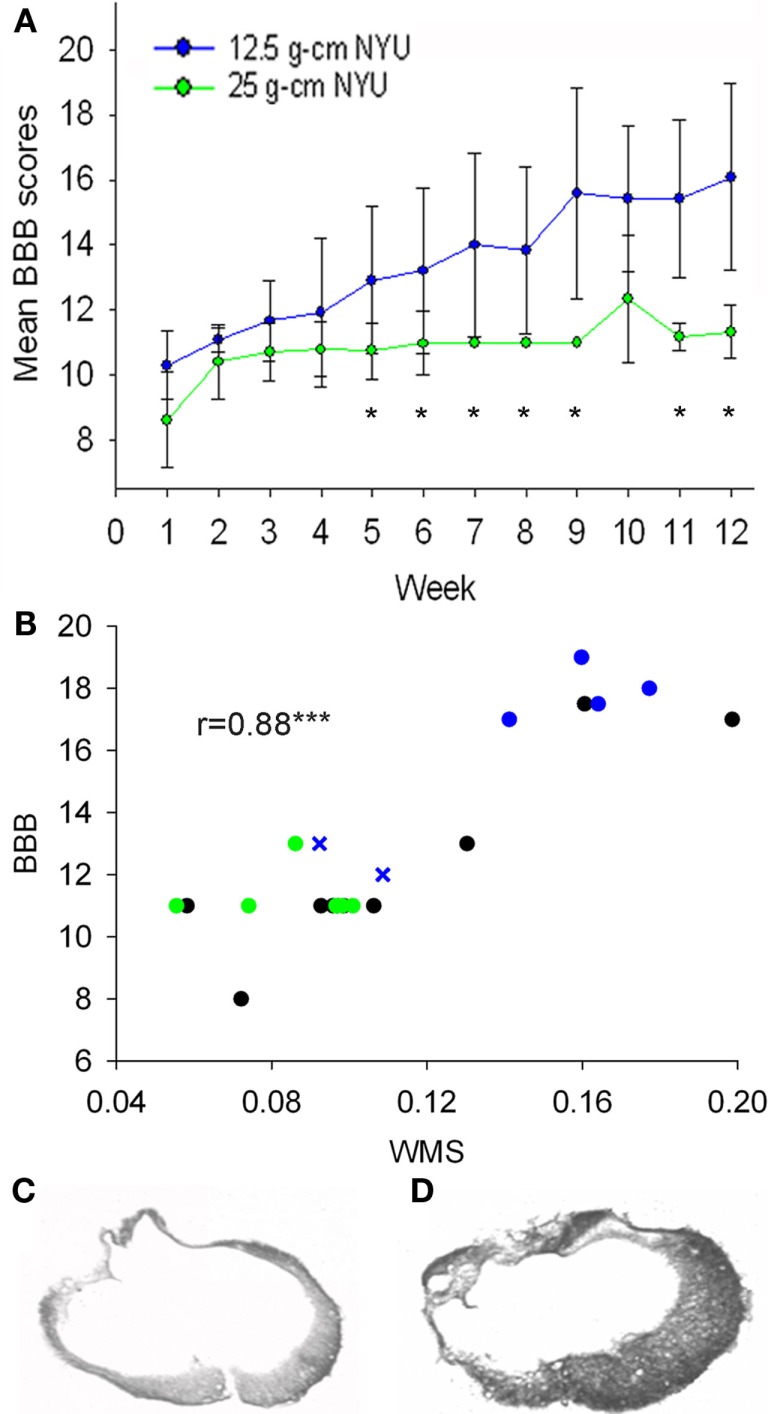
**Injuries were characterized using BBB scores to assess hindlimb locomotor function and white matter sparing (WMS) at epicenter using EC stain**. **(A)** BBB scores of groups that received 12.5 g cm (blue) or 25 g cm (green) NYU injuries. Starting at week 5, a significant difference was observed between the two injury severities. Both groups were significantly different (*p* < 0.05) from controls (not shown) at all time points. **(B)** White matter sparing at epicenter (*x* axis) plotted vs. BBB score. Black dots represent 6 week values. Green dots represent 12 week, 25 g cm injured animals. Blue x represent the two animals from the 12-week, 12.5 g cm group with the lowest BBB scores within the group. Blue dots represent the four animals from the 12-week, 12.5 g cm group with the highest BBB scores within the group. **(C)** Image taken from a 12.5 g cm contused animal showing a laterally-symmetrical injury pattern at the epicenter. Note the difference from **(D)**, which was taken from an animal that also received a 12.5 g cm spinal cord contusion but which yielded an asymmetrical injury at epicenter. *p < 0.05, ***p < 0.001.

To assess the degree of injury severity, we characterized SC injuries based on two parameters; behavior as measured by BBB, and the amount of SWM at the epicenter after staining with eriochrome cyanin (Rabchevsky et al., 2007). BBB scores were significantly greater in the 12.5 g cm injury groups than the 25 g cm groups beginning at week 5 (Figure 1A). These differences in behavior were reflected in the amount of SWM, as the 25 g cm groups had 8.5% (±1.8%) and the 12.5 g cm groups had 13.9% (±3.6%) SWM at the epicenter. In accord with prior literature (Basso et al., 1996; Schucht et al., 2002; Magnuson et al., 2005), a significant correlation (*r* = 0.88, *p* < 0.001) was observed between white matter sparing at epicenter and BBB scores (Figure 1B). BBB scores of the 12.5 g cm group showed a high degree of variability and continued to increase between 6 and 12 weeks instead of reaching a plateau. Within this group, two animals had BBB scores consistent with the range observed in previous literature (Basso et al., 1996; Agrawal et al., 2010; 12 and 13) and four animals that had higher BBB scores than expected for this injury severity (mean 17.9) at 12 weeks post injury. We considered that these results may be due to both greater amount of SWM and/or asymmetry of the lesion (Figures 1C,D). Indeed, of the four animals whose BBB scores continued to increase, all had a greater amount of SWM (mean 16.1% for four animals with higher BBB scores, 10.1% for two animals with lower BBB scores), and all had asymmetrical injuries (arbitrarily defined as more than 4% greater SWM on one side vs. the other). Animals with the lower BBB scores in the 12.5 g cm group did not represent statistical outliers (Grubbs outlier test). Separate statistical analyses of gene expression were performed with the exclusion of the two animals whose BBB scores did not continue to increase and the results generally did not differ from those found when all six animals were considered together. The lone exception was the results for expression of one Trk receptor in the SC, which is noted below. We thus consider all six animals together in the group in all subsequent figures and analyses of mRNA expression.

### Expression of Trk receptors in the DRG

**Figure 2:**
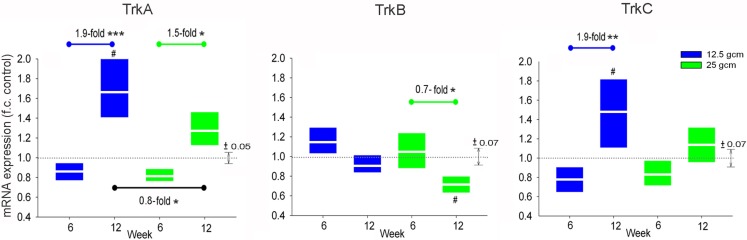
**mRNA expression of Trk receptors is altered in L4/5 DRG at 6 and 12 weeks after receiving either 12.5 g cm (blue) or 25 g cm (green) NYU contusion injury to spinal cord at vertebral level T9**. Fold-change (FC) is reported as change of 12 week relative to 6 week time points in all figures. Black bar on TrkA reports fold-change (fc) of 25 g cm at 12 weeks relative to 12.5 g cm at 12 weeks. X axis denotes weeks post injury. White lines in box-plots indicate group mean. Dotted gray lines indicate expression level of controls (normalized to 1), with ±SEM indicated by the vertical arrows at right end of the control line. #*p* < 0.05 vs. control, *p < 0.05, **p < 0.01, ***p < 0.001.

One purpose of this study was to determine whether these different contusion severities result in a differential transcriptional response of neurotrophins and their Trk receptors in lumbar sensorimotor circuits. Hence, we sought to determine the expression level of Trk receptors in the DRG 6 and 12 weeks after our two severities of contusion injury. Expression of TrkA, TrkB, and TrkC each differed significantly between the 6- and 12-week groups, with the magnitude and direction of difference depending on receptor type and injury severity. Expression of TrkA mRNA in DRG from the 12-week group at both injury severities was significantly greater than that in DRG from the corresponding 6 week group. Expression of TrkA in DRG from the 12-week group that received 12.5 g cm injury was also elevated relative to the control groups. We also observed a difference in TrkA expression between injury severities at the 12-week time point. Similar to TrkA, expression of TrkC mRNA in DRG from the 12-week group was greater than that in DRG from the corresponding 6 week group at both injury severities, but the difference only reached significance in the 12.5 g cm animals. Unlike the findings for TrkA, we detected no significant difference in TrkC expression between DRG from the 12.5 g cm group and from the 25 g cm group at the 12 week time point. Expression of mRNA for TrkB in DRG at 12 weeks after 25 g cm injury was significantly lower than in DRG from both the 6-week SCI and control groups. No significant difference in TrkB expression was observed between injury severities at 6 or 12 week time points in the 12.5 g cm injury group (Figure 2).

### Expression of neurotrophins in the DRG

**Figure 3:**
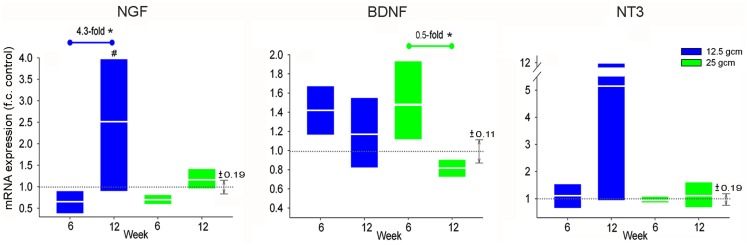
**mRNA expression of Neurotrophins is altered in L4/5 DRG at 6 and 12 weeks after receiving either 12.5 g cm (blue) or 25 g cm (green) NYU contusion injury to spinal cord at vertebral level T9**. Fold-change (fc) is reported as change of 12 week relative to 6 week time points in all figures. X axis denotes weeks post injury. White lines in box-plots indicate group mean. Dotted gray lines indicate expression level of controls (normalized to 1), with ±SEM indicated by the vertical arrows at right end of the control line. #*p* < 0.05 from control. *p < 0.05, **p < 0.01, ***p < 0.001.

As with TrkA, NGF mRNA expression in DRG from the 12.5 g cm injury severity group was significantly greater in the 12-week group than in both the 6-week and control groups. However, no significant changes in NGF expression were observed between survival time groups in the 25 g cm injury severity group. As with TrkB, BDNF expression in the 12-week 25 g cm group was significantly less than in the 6-week 25 g cm group, but did not differ from the control group (Figure 3). No other differences were observed in BDNF expression levels. There was a large increase in the mean expression of NT3 in DRG from the 12-week, 12.5 g cm injury group, however due to high variance no significant differences were observed from 6 to 12 weeks.

### Expression of Trk receptors in the spinal cord

**Figure 4:**
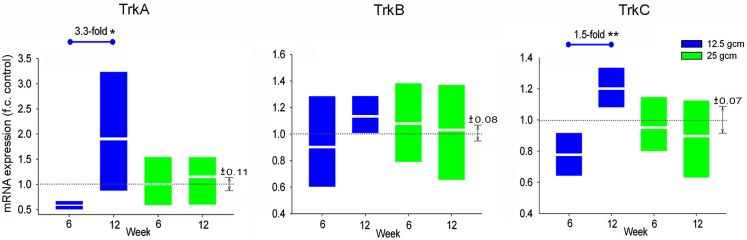
**mRNA expression of Trk receptors is altered in L4/5 spinal cord at 6 and 12 weeks after receiving either 12.5 g cm (blue) or 25 g cm (green) NYU contusion injury to spinal cord at vertebral level T9**. Fold-change (fc) is reported as change of 12 week relative to 6 week time points in all figures. *X* axis denotes weeks post injury. White lines in box-plots indicate group mean. Dotted gray lines indicate expression level of controls (normalized to 1), with ±SEM indicated by the vertical arrows at right end of the control line. #*p* < 0.05 from control. *p < 0.05, **p < 0.01, ***p < 0.001.

Expression levels of mRNA for neurotrophin receptors TrkA, TrkB, and TrkC were assessed from samples of lumbar SC (L4/5). In the groups that received a 12.5 g cm injury, the level of TrkA in SC from the 12-week group was significantly greater than that from the 6-week group, whereas there was no significant difference between the two post-SCI times in the 25 g cm injury group. Like TrkA, the level of TrkC in SC from the 12-week 12.5 g cm group was significantly greater than that from the 6-week group, with no significant difference between the two post-SCI times in the 25 g cm injury group. No significant changes in TrkB expression levels were detected between any groups (Figure 4).

### Expression of neurotrophins in the spinal cord

**Figure 5:**
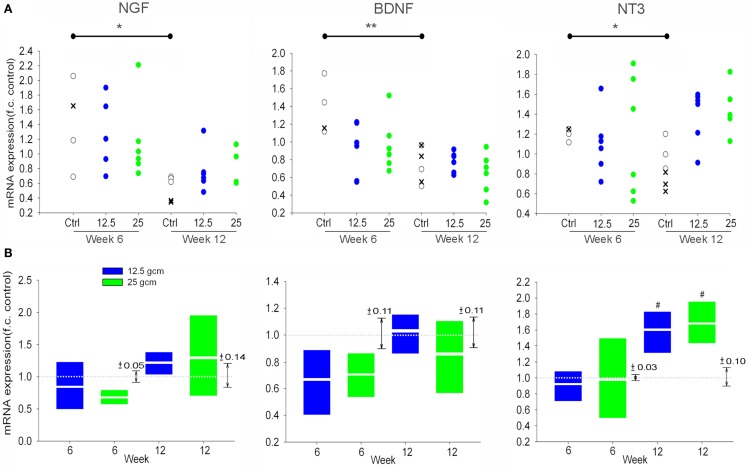
**(A)** Scatterplots of Neurotrophin mRNA expression in L4/5 SC at 6 and 12 weeks after receiving either 12.5 g cm (blue) or 25 g cm (green) NYU contusion injury to spinal cord at vertebral level T9, *relative to the unified control group as was done for the other data*. *x*’s represent mRNA expression of age-matched naive animals. Open circles represent expression of age-matched laminectomy control animals. *p < 0.05, **p < 0.01 **(B)** mRNA expression of Neurotrophins in L4/5 SC at 6 and 12 weeks after receiving either 12.5 g cm (blue) or 25 g cm (green) NYU contusion injury to spinal cord at vertebral level T9, *relative to their respective age-matched control groups*. *X* axis denotes weeks post injury. White lines in box-plots indicate group mean. Dotted gray lines indicate expression level of controls (normalized to 1), with ±SEM indicated by the vertical arrows to the right of each time point pair. #*p* < 0.05 from control.

The results for neurotrophins in the SC are displayed differently from the data regarding expression levels of neurotrophins and Trk receptors in the DRG, and Trk receptors in the SC. In the latter assessments, the expression of neurotrophins and trks did not differ between the 6- and 12-week control animals. Thus, those data were analyzed and presented relative to the mean and variation of a single unified control group. This allowed us to simultaneously assess the effect of both injury severity and survival time on gene expression. For the neurotrophin genes in SC, however, expression differed significantly between the 6- and 12-week control groups (Figure 5A). We first analyzed these gene expression data exactly as was done for the other tissues – comparing each injury severity and survival time to the mean and variation of a single unified control group – but for the sake of clarity we have presented the data from the individual animals in each group. Caution must be exercised when considering the expression data for the experimental groups in this analysis (Figure 5A) because of the use of a unified control group – i.e., these data were generated exactly as were the other expression values, but are relative to a unified control group that, in this case, is not a suitable control group. We found decreases between our 6 and 12 week control groups in expression levels of NGF, BDNF, and NT3 in the SC in the absence of SCI. It is worth noting that our quality control measures were repeated for these samples, but the assessments remained the same. In ruling out technical issues and variability due to the necessity of using animals from different litters, a single factor appears to account for the altered expression levels in the control groups, that being age.

Because the gene expression differed between the 6- and 12-week control groups, we cannot incorporate the temporal characteristic of the experimental design in our assessment of neurotrophin expression in SC. We are limited to analyzing the effect of injury severity on gene expression within each separate survival time group, where the data from experimental groups is expressed relative to the time-matched control group only (Figure 5B). Considered in this way, SCI itself did not significantly influence expression of any neurotrophin at any time considered, with the exception of NT3 at 12 weeks post-SCI. At this time, NT3 was elevated relative to the time-matched control group, with no effect of injury severity.

### Relationship of transcriptional assessments to functional and anatomical assessments

Our experimental design was intended to embrace the variability that exists with models of contusive SCI in that we also examined whether a statistical correlation existed between expression levels of each transcript and BBB or white matter sparing on an animal by animal basis. We observed no statistically significant correlation between the expression levels of the transcripts and BBB score or white matter sparing.

### Coordinated expression of neurotrophins and Trk receptors in DRG and spinal cord 12 weeks post injury

**Figure 6:**
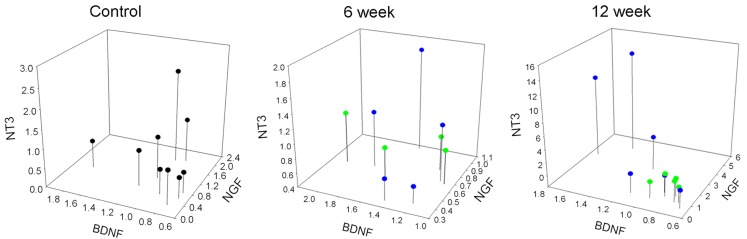
**Correlated expression of neurotrophins in DRG emerges at chronic time points**. Values represent fold-change of the individual animals vs. mean of control group. Blue dots represent animals with 12.5 g cm injuries. Green dots represent animals with 25 g cm injuries.

**Figure 7:**
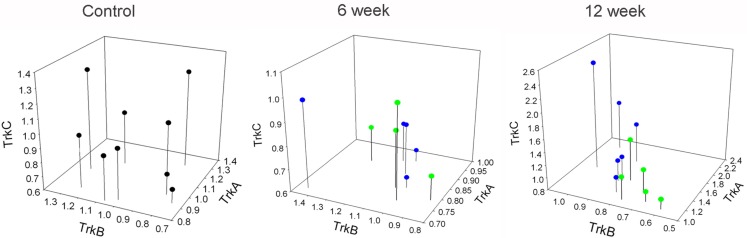
**Correlated expression of Trk receptors in DRG emerges at chronic time points**. Values represent fold-change of the individual animals vs. mean of control group. Blue dots represent animals with 12.5 g cm injuries. Green dots represent animals with 25 g cm injuries.

To further characterize the relationship between the neurotrophins and their receptors in lumbar DRG and SC, we analyzed the expression levels of neurotrophins and Trk receptors relative to each other, and without respect for injury severity. In the SC, the only significant relationship was that of TrkB and TrkC in the control and 6 week groups. No relationship was found between any other expression levels at any time points in the SC (Table 3). In the DRG, there was a relationship between NGF and NT3 in all groups. In the 6-week groups the only other significant correlation observed was between BDNF and TrkB. After 12 weeks there was a significant correlation in the expression levels of all neurotrophins in the DRG, a relationship that existed for the Trk receptors as well (Table 4). Additionally, a significant correlation was observed between expression levels of neurotrophins and their cognate Trk receptors at 12 week time points (Table 4). This coordinated expression pattern occurred in all animals independent of injury severity (Figures 6 and 7). The reliability of this statistical assessment is enhanced by our performing the reverse-transcription for all samples with the same reagents, a procedure which reduces the cross-sample variability.

**Table 3:** **Correlations between expression of mRNA for trk receptors in spinal cord**.

	Control		6 week		12 week	
	*r*-Value	*p*-Value	*r*-Value	*p*-Value	*r*-Value	*p*-Value
TrkA vs. TrkB	0.47	0.2	0.25	0.46	0.19	0.56
TrkA vs. TrkC	0.42	0.26	0.21	0.53	0.4	0.19
TrkB vs. TrkC	0.75	**0.01**	0.76	**0.004**	0.44	0.16

*Data in bold are statistically significant*.

**Table 4:** **Correlations between expression of mRNA for neurotrophins, Trk receptors, and cognate pairs in DRG**.

	Control		6 week		12 week	
	*r*-Value	*p*-Value	*r*-Value	*p*-Value	*r*-Value	*p*-Value
NGF vs. BDNF	0.20	0.61	0.15	0.69	0.84	**0.001**
NGF vs. NT3	0.72	**0.03**	0.87	**0.003**	0.92	**0.00006**
BDNF vs. NT3	0.10	0.80	0.33	0.38	0.88	**0.0004**
TrkA vs. TrkB	0.04	0.90	0.005	0.989	0.89	**0.0007**
TrkA vs. TrkC	0.56	0.11	0.61	0.08	0.79	**0.006**
TrkB vs. TrkC	0.40	0.28	0.38	0.31	0.78	**0.004**
NGF vs. TrkA	0.65	0.06	0.55	0.13	0.88	**0.0004**
BDNF vs. TrkB	0.45	0.22	0.69	**0.04**	0.77	**0.005**
NT3 vs. TrkC	0.57	0.11	0.61	0.08	0.77	**0.006**

*Data in bold are statistically significant*.

### Bioinformatic analysis of neurotrophin and Trk receptor gene regulation

In light of the apparent coordinated expression of neurotrophins and trk receptors in DRG at 12 weeks after SCI, we used bioinformatic analyses to examine some possible mechanisms that may be at play. In order to assess possible coordination of regulation via gene promoters, we retrieved from the TransFac database (Wingender et al., 2000; Wingender, 2008; gene-regulation.com) all transcription factors (TFs) known/predicted to bind to (1) the annotated promoter region or (2) the sequence 1 kb upstream of the annotated translation start-site if the annotated promoter was less than 1 kb, of all six genes examined here. For this procedure the RGSC 5.0/rn5 (March 2012; genome.ucsc.edu) rat genome assembly was used and all sequences and locations are relative to this assembly (Table 5). These broad results were filtered for those TFs with annotations indicating expression in nervous tissue, and results for different transcript entries for the same gene were pooled. TrkC was the only gene to lose all TFs in this filtering process, reflecting the fact that the assembled sequence upstream of the TrkC gene has numerous stretches of undefined bases, and that the annotated promoter is very short. In spite of this, numerous TFs remained for three or more genes, and four TFs remained for all genes (except trkC) – cyclic AMP response element binding protein (CREB), MafB, NeuroD, and Pax3 (Table 6).

**Table 5:** **Genomic coordinates used for Bioinformatic analyses**.

Gene	RefSeq	Chromosome	CDS Beg	CDS End	Strand	5′UTR Beg	5′UTR End	3′UTR Beg	3′UTR End
Ntrk1 (TrkA)	NM_021589	chr2	206548727	206565310	–	206565311	206570310	206543727	206548726
Ntrk2 (TrkB)	NM_012731.2	chr17	8158054	8463473	–	8463474	8468473	8153054	8158053
Ntrk2 (TrkB)	NM_001163168.1	chr17	8340214	8463473	–	8463474	8468473	8335214	8340213
Ntrk2 (TrkB)	NM_001163169	chr17	8389944	8463473	–	8463474	8468473	8384944	8389943
Ntrk3 (TrkC)	NM_001270655.1	chr1	140868438	141239903	–	141239904	141244903	140863438	140868437
Ntrk3 (TrkC)	NM_001270656.1	chr1	140868438	141239903	–	141239904	141244903	140863438	140868437
Ntrk3 (TrkC)	NM_019248.1	chr1	140868438	141239903	–	141239904	141244903	140863438	140868437
NGF	NM_001112698.1	chr2	224368770	224369496	+	224363770	224368769	224369497	224374496
NGF	NM_013609.2	chr2	224362515	224369496	+	224357515	224362514	224369497	224374496
BDNF	NM_001270631	chr3	107418271	107419021	+	107413271	107418270	107419022	107424021
BDNF	NM_001270632	chr3	107418271	107419021	+	107413271	107418270	107419022	107424021
BDNF	NM_001270633	chr3	107418271	107419021	+	107413271	107418270	107419022	107424021
BDNF	NM_001270634	chr3	107418271	107419021	+	107413271	107418270	107419022	107424021
BDNF	NM_001270635	chr3	107418271	107419021	+	107413271	107418270	107419022	107424021
BDNF	NM_001270636	chr3	107418271	107419021	+	107413271	107418270	107419022	107424021
BDNF	NM_001270637	chr3	107418271	107419021	+	107413271	107418270	107419022	107424021
BDNF	NM_001270638	chr3	107418271	107419021	+	107413271	107418270	107419022	107424021
BDNF	NM_001270630	chr3	107390677	107419021	+	107385677	107390676	107419022	107424021
BDNF	NM_012513	chr3	107371964	107419021	+	107366964	107371963	107419022	107424021
Ntf3 (NT3)	NM_031073	chr4	225639116	225705803	–	225705804	225710803	225634116	225639115
Ntf3 (NT3)	NM_001270869	chr4	225639116	225705803	–	225705804	225710803	225634116	225639115
Ntf3 (NT3)	NM_001270868	chr4	225639116	225675123	–	225675124	225680123	225634116	225639115
Ntf3 (NT3)	NM_001270870	chr4	225639116	225639893	–	225639894	225644893	225634116	225639115

**Table 6:** **Transcription factor binding sites for neurotrophin and trk receptor genes**.

TF Binding-site name	HGNC symbol	TrkA	NGF	TrkB	BDNF	TrkC	NTF3
AhR	AHR	x		x	x		
AhR: Arnt					x		
AP-1	FOS; FOSB; JUN; JUND		x		x		
AP-2	TFAP2A	x	x	x	x		
AR	AR			x	x		
Arnt	ARNT			x			
ATF	ATF	x		x			
ATF2	ATF2	x	x	x	x		
ATF2: c-Jun			x				
Brn-2	POU3F2				x		
C/EBP	CEBPA, B, D, E, G, Z	x			x		
CAR	NR1I3	x					
c-Ets-2	ETS2		x				
c-Jun	JUN	x		x			
c-Myc: Max		x					
COUP-TF1	NR2F1		x		x		x
CREB	CREB1	x	x	x	x		x
CREB, ATF					x		
CREM	CREM	x		x			
DEC	BHLHE40	x	x		x		
E2A	TCF3			x			
Ebox	TCF3; MYOD1; MYOG			x			
ER-alpha	ESR1				x		x
Ets	ETS1, 2; ETV1, 2, 3, 4, 5, 6, 7		x		x		x
Foxj1	FOXJ1						x
FOXO1	FOXO1						x
GATA-3	GATA-3				x		x
GR	NR3C1		x	x	x		
HES1	HES1			x			x
HOXA5	HOXA5		x				
HOXB8	HOXB8	x					
KROX	EGR1, 2; ZNF22; ZBTB7B				x		
MAF	MAF		x	x	x		x
MAFB	MAFB	x	x	x	x		x
Max	MAX	x			x		x
MEF-2	MEF-2A						x
MEF-2C	MEF-2C				x		
Myc	MYC	x			x		
Neuro D	NEUROD1	x	x	x	x		x
NFAT1	NFATC2	x	x		x		x
NF-AT4	NFATC3	x	x		x		x
NF-kappaB	NFKB1		x				
NKX2B	NKX2-2		x				
NRSF	REST		x				
NURR1	NR4A2	x	x		x		
Oct-1	POU2F1		x		x		
Octamer	POU family of proteins		x		x		
Oct-x	STAT1		x				
p53	TP53						x
Pax3	PAX3	x	x	x	x		x
Pax6	PAX6						x
Pax8	PAX8		x	x	x		
Pbx1	PBX1		x		x		x
POU6F1	POU2F1				x		
POUF2F1	POU6F1		x				
PPARgamma	PPARG			x			
PPARgamma: RXR-alpha				x			
PXR	NR1I2	x					
RXR-alpha	RXRA			x	x		
SF1	SF1						x
SMAD	MADH family of proteins				x		x
Smad3	SMAD3				x		x
Sox1	SOX1				x		x
Sox2	SOX2		x				
Sp1	SP1	x	x		x		x
SRF	SRF		x				
Sry	SRY		x		x		x
STAT	SOAT1		x		x		x
STAT1	STAT1		x				
STAT3	STAT3		x		x		
Tax	CNTN2			x			
Tax/CREB					x		x
Tbp	TBP			x	x		x
TCF4	TCF4	x					
Tcfap2a	TFAP2A				x		
Tcfap2b	TFAP2B				x		
Tst-1	CCDC6		x				
USF	USF1				x		
USF2	USF2				x		
VDR	VDR	x					
VDR, CAR, PXR					x		x

*Entries with transcription factors separated with a “:” have binding sites situated such that they act in a cooperative fashion, rather than independent from each other. Entries with transcription factors separated by a “,”share binding sites or have binding sites situated near each other such that the factors act in competition with each other*.

Another possible means of regulating the levels of mRNA is by micro-RNA (miRNA), which can influence the stability and/or turn-over rate of transcripts, among other effects (e.g., Kosik and Krichevsky, 2005). In order to assess possible coordination of regulation via miRNA, we retrieved from the TargetScan database those miRNA-binding sites that are conserved between human and rat neurotrophin and trk genes (TargetScanHuman release 6.2, e.g., Grimson et al., 2007; targetscan.org; Table 7). Although numerous miRNA species were retrieved, none were shared across any of the neurotrophin and trk genes.

**Table 7:** **Micro-RNA binding sites for neurotrophin and trk receptor genes**.

Gene symbol	miRNA symbol
Ntrk1 (TrkA)	n/a
Ntrk2 (TrkB)	rno-miR-325p
Ntrk2 (TrkB)	rno-mir-211
Ntrk2 (TrkB)	rno-miR-204
Ntrk3 (TrkC)	rno-mir-128
Ntrk3 (TrkC)	rno-miR-466b
Ntrk3 (TrkC)	rno-miR-297
Ntrk3 (TrkC)	rno-miR-3592
NGF	rno-let-7e
NGF	rno-let-7d
NGF	rno-let-7b
NGF	rno-let-7c
NGF	rno-let-7a
NGF	rno-miR-98
NGF	rno-let-7f
NGF	rno-let-7i
BDNF	rno-miR-10a-5p
Ntf3 (NT3)	rno-miR-222
Ntf3 (NT3)	rno-miR-221

## Discussion

### Expression and function of neurotrophins and Trks

Spinal cord injury engenders a host of changes to both the central and peripheral nervous system, indeed for the entire organism, with residual functional capacity that is largely dependent on the location and severity of the injury. A variety of different approaches have been used in efforts to re-establish function, including enhancement of regeneration across the injury site (e.g., Bregman et al., 2002; Moon and Bunge, 2005; Sharma et al., 2012; Smith et al., 2012) and plasticity of intact circuits below the level of the lesion (e.g., Edgerton et al., 2004; Boulenguez and Vinay, 2009; Rossignol and Frigon, 2011). One means for achieving plasticity of intact circuits is through activity-dependent reorganization of inputs (e.g., Edgerton et al., 2004). This phenomenon has been described in studies of both animal (reviewed in Edgerton et al., 2008) and human (reviewed in Harkema, 2008) of SCI. Neurotrophins have been implicated as having a role in such changes (Hutchinson et al., 2004; Boyce et al., 2007, 2012; Côté et al., 2011). However, activity-dependent changes in the capacity for locomotion often manifest at times later than those examined in studies of post-SCI expression of neurotrophins and trk receptors (De Leon et al., 1998, 1999; Table 3). Indeed, the dynamic period of spontaneous locomotor recovery generally lasts for approximately 6 weeks after SCI, a time well beyond most prior studies (Table 3). In addition to a likely role in locomotor function, neurotrophin signaling is implicated in pathologic outcomes of plasticity such as post-SCI pain and autonomic dysreflexia (e.g., Brown and Weaver, 2012). The role of neurotrophin signaling has principally been examined in terms of initiation of these conditions in the near-term after SCI in animal models (Krenz et al., 1999; Marsh et al., 2002; Cameron et al., 2006), as opposed to later-phase initiation or maintenance. The regulation we have demonstrated at extended time points may provide new rationale for examining the role of neurotrophin signaling in later stages of these conditions.

Neurotrophins exert modulatory effects on cellular physiology through activation of their cognate Trk receptors (Lindsay, 1996; Patapoutian and Reichardt, 2001; Huang and Reichardt, 2003). In the DRG, expression of neurotrophin receptors is restricted to specific populations of cells. Generally, TrkA is expressed in neurons with small soma size, TrkB in neurons with intermediate size, and TrkC in neurons with large soma size; populations of TrkA and TrkC expressing neurons remain largely separate, whereas TrkB is co-expressed in overlapping populations of TrkA and TrkC positive cells (Mu et al., 1993; McMahon et al., 1994; Wright De, 1995; McMahon, 1996; Phillips and Armanini, 1996). Trk receptors are not ubiquitous, however, as there is a large subpopulation of small diameter DRG neurons which do not express any of the Trk receptors or the low-affinity neurotrophin receptor p75 in the adult (McMahon et al., 1994; Molliver and Snider, 1997; Bennett et al., 1998). In the mammalian SC, TrkA is expressed in second order nociceptors of the dorsal horn, TrkB has a broad pattern of expression which overlaps with both TrkA and TrkC expression, and TrkC is expressed in neurons of the intermediate and ventral horn (e.g., Duberley et al., 1997; Curtis et al., 1998; Schober et al., 1999; Copray and Kernell, 2000; Liebl et al., 2001; Allen Institute for Brain Science, 2009).

As long as 6 weeks after SC transection injury, the number of cells expressing TrkA and TrkB protein in L1 and L6/S1 DRG (containing bladder afferents) increases over controls, though the numbers of cells expressing these genes does not significantly change in L4/5 DRG (Qiao and Vizzard, 2002, 2005). Our analysis of Trk expression, which was also performed in L4/5 DRG and included a 6-week post-SCI time point, found no significant change in the trkA or trkB mRNA levels for either severity of contusion injury, in agreement with the prior work. In intact sensory and sympathetic ganglia of the adult rat, NGF and NT3 (as well as TrkA, full length TrkB, and TrkC), localize exclusively to neurons; BDNF and the truncated isoform of TrkB are expressed more extensively, however, localizing to neuronal cells and some glial and satellite cells (Wetmore and Olson, 1995). These observations are consistent with the notion that full length Trk expression predominantly occurs in neurons, though since the latter study was performed with intact animals, we cannot exclude the possibility that our injuries potentially resulted in expression in other cell types. Indeed, there are numerous reports of trk receptor expression by non-neuronal cells. In particular Schwann cells can express trks, as can cancer cells (e.g., Funakoshi et al., 1993; Tacconelli et al., 2005; Hess et al., 2007; Jin et al., 2011). Further, neurotrophins are often expressed in non-neuronal cells, most notably by cells outside the nervous system where they influence both developmental and adult processes (e.g., Lewin, 1996; Petruska and Mendell, 2004).

Previous assessments of changes in neurotrophin/Trk receptor expression levels after SCI have typically focused at time points of less than 6 weeks. BDNF expression increases up to 2 weeks after injury in the SC after thoracic transection and crush injury (Hayashi et al., 2000; Li et al., 2007), though both increases and decreases in expression have been reported after hemisection during a similar time period post injury (Gulino et al., 2004; Qin et al., 2006). Expression levels of NGF and NT3 in the cord increase for up to 3 weeks after SCI (Hayashi et al., 2000; Li et al., 2007). In another study, NGF and BDNF transcripts were found to increase up to 4 days following injury in the adult cord, however, by 2 weeks post injury all neurotrophins were expressed at levels similar to that of control (Nakamura and Bregman, 2001; Widenfalk et al., 2001), suggesting expression decreases after an early increase, though these studies used different injury models. Trk mRNA expression is downregulated acutely in the SC at and around the injury site after contusion (Liebl et al., 2001; Hajebrahimi et al., 2008), however by 6 weeks expression levels are not different from control (Liebl et al., 2001). However, after SC transection TrkC has been shown to increase after 2 weeks (Qian et al., 2011). Similarly, in a recent study assessing mRNA and protein changes after transection at 10 and 31 days post injury, whole SC TrkB mRNA was elevated at 10 days post injury, and whole SC NT3 and TrkB protein was elevated at 31 days post injury, with expression differences also observed depending on the location within the parenchyma of the SC (Keeler et al., 2012). Table 1 summarizes the findings of recent experiments to facilitate comparison of these results.

We found TrkA expression increases in both the DRG and SC of animals after contusion in a manner that was dependent on injury severity. This finding is of particular interest with regards to the functions of NGF and TrkA. NGF plays a well-defined role in sensitization of nociceptive afferent neurons (e.g., Shu and Mendell, 1999, 2001; Galoyan et al., 2003; Zhu et al., 2004b). Nociceptive DRG neurons undergo changes after SCI, including development of spontaneous activity (Bedi et al., 2010) and an enhanced intrinsic growth promoting state (Bedi et al., 2012). Such changes in anatomical and physiological properties of nociceptors may contribute to development of conditions such as autonomic dysreflexia (e.g., Marsh et al., 2002). TrkA antagonists prevent the sensitization (thermal and mechanical hyperalgesia) normally induced by partial nerve injury (Ma et al., 2010), and antagonism of TrkA signaling has been effective for controlling human pain (Mantyh et al., 2011). Hence, elevation in the levels of TrkA and NGF in response to contusive injury could play a role in some of the maladaptive processes after incomplete SCI.

TrkB activation has also been implicated in hypersensitivity to nociceptive input and sensitization of nociceptors (Kerr et al., 1999; Shu et al., 1999; Garraway et al., 2003). However, after either SC transection or contusion injury, BDNF induced facilitation of afferent responses in lamina II of the dorsal horn is significantly reduced (Garraway et al., 2005; Garraway and Mendell, 2007). Our results could suggest a mechanism for those physiological observations. In addition to TrkB expression in populations of second order nociceptive neurons (Schober et al., 1999), it is expressed robustly throughout the interneuronal circuitry, and also co-expressed along with NT3 in motoneurons (Buck et al., 2000), a finding corroborated in humans (Josephson et al., 2001). Notably, BDNF administration to the injured SC can improve locomotor outcomes, however because of its influence on nociceptive circuitry its therapeutic utility may be limited (Boyce et al., 2012).

In DRG, TrkC is present on medium to large diameter muscle spindle afferents that make monosynaptic connections with motoneurons and cutaneous low threshold mechanoreceptors (Klein et al., 1994; Oakley et al., 1997; Josephson et al., 2001) in the intermediate and ventral horns of the SC. NT3, likely acting via TrkC, exerts a modulatory effect on sensorimotor circuits in both intact (Petruska et al., 2010) and injured preparations (Mendell et al., 2001; Arvanian et al., 2003; Arvanian et al., 2006a,b; García-Alías et al., 2011; Schnell et al., 2011). Locomotor training after SCI is associated with increased expression levels of TrkB and TrkC agonists in rats (Hutchinson et al., 2004; Côté et al., 2011). In addition, co-administration of both BDNF and NT3 to the injury site has been shown to improve hindlimb locomotion after transection in both rats (Blits et al., 2003) and cats (Boyce et al., 2007). Taken together, these findings suggest a potential role for Trk activation in modulation of lumbar sensorimotor circuitry in both intact and injured animals.

The apparent age-related regulation of NGF, BDNF, and NT3 in non-injured SC was unexpected and we made significant efforts to identify possible technical and sampling issues. While those factors that often account for variability did not satisfactorily account for the expression patterns we observed, the single factor of age did appear to fully account for the differences. Expression of the neurotrophins has been examined in the context of embryonic and postnatal development and in aging (e.g., Timmusk et al., 1994; Nosrat, 1998; Bergman et al., 2000). However, to the best of our knowledge, there has been no systematic assessment of the regulation of the neurotrophins at such late postnatal times. If it is indeed borne out that neurotrophins are regulated in the SC over a long postnatal time course, this must be taken into account when designing experiments that may be influenced by the natural progression of this expression.

### Coordinated expression of neurotrophins and Trks

Twelve-weeks after injury a coordinated expression pattern existed among the levels of all neurotrophins and Trk receptors regardless of injury severity, and also between the neurotrophins and their cognate Trk receptors in the DRG (Tables 1 and 2, Figures 5 and 6), a relationship that was not evident at 6 weeks post-SCI. Although there are reports of smaller groups of neurotrophins and/or Trks being regulated in a coordinated fashion (e.g., Widenfalk et al., 1999), to our knowledge this degree of coordination has not been reported, and the mechanism is unclear. One obvious possibility is a feedback/feed-forward relationship between some/all of these genes, and these sorts of relationship do exist (e.g., Michael et al., 1997; Wyatt et al., 1999; Gibbons and Bailey, 2005).

Neurotrophin dependent neurotrophin expression has been demonstrated *in vitro* in NIH3T3 and PC12 cells (Canossa et al., 1997; Mallei et al., 2004), hippocampal neurons (Canossa et al., 1997), and cerebellar granule neurons (Leingärtner et al., 1994). *In vivo*, intrathecal administration of NT3 to intact adult animals for 1 week results in reduced expression of TrkA protein in the DRG, but has no effect on levels of TrkC (Gratto and Verge, 2003). After unilateral axotomy, sub-cutaneous administration of exogenous NT3 similarly causes a decrease in TrkA on the side contralateral to the injury. This contrasts to the increase in TrkA expression seen on the side ipsilateral to the injury; the effect of NT3 on expression levels of TrkB and TrkC however is not affected by injury in this paradigm, as levels of these transcripts show increased expression up to 4 weeks post axotomy in both ipsi- and contra-lateral DRG (Kuo et al., 2007). Such coordinated expression patterns could potentially result from changes at the epigenetic level or from interactions between the different transcription factors associated with expression of specific transcripts. During development, Runx1 and Runx3 transcription factors play essential roles in cell fate determination of nociceptive (Chen et al., 2006) and proprioceptive (Inoue et al., 2002) neurons, respectively. Much attention regarding transcriptional regulation of neurotrophin expression in the mature nervous system has been given to BDNF, due to its role in activity-dependent mechanisms during long-term potentiation (LTP). Such investigations have revealed several important transcriptional regulators including, CREB, calcium-responsive transcription factor (CaRF), and methyl CpG-binding protein 2 (MeCP2; Tao et al., 1998, 2002; Chen et al., 2003; Reichardt, 2006). Such findings may facilitate future efforts to determine the mechanisms regulating the expression of the neurotrophins and Trk receptors in the injured adult SC and sensory ganglia.

The lack of any TFs for trkC after the filtering process is more a reflection of the relative amount of data available than reality. The filtering step in the bioinformatic analysis involved the use of annotations, which, valuable though they are, have inherent limitations. Certainly there are published data regarding factors involved in regulating the expression of trkC, particularly Runx3 (Levanon et al., 2002), Brn3a/Pou4f1 and Runx1 (Zou et al., 2012), and REST/NRSF (Nakatani et al., 2005).

In spite of the lack of any result related to TrkC, four TFs did emerge as possibly interacting with all of the remaining genes. The majority of published information related to these genes and their involvement in regulation of neurotrophins and Trks is in the context of development or cancer. This does not imply that they function exclusively in those contexts, but only that those contexts are the most studied. We could not identify any studies examining Pax3, NeuroD, or MafB in SC or DRG in the context of SCI. Maf has been studied in relation to neurodegeneration (Kobayashi et al., 2011) and in stress (Machiya et al., 2007). Pax3 was studied in relation to nerve injury, where it was found to not be regulated (though this does not imply it not being active; Vogelaar et al., 2004).

There are studies examining CREB in SC (Crown et al., 2005, 2006; Yu and Yezierski, 2005; Yune et al., 2008) or DRG (Qiao and Vizzard, 2005) in the context of SCI, with the latter study examining TrkA, TrkB, and CREB, though not in direct relation to each other. Interestingly, the expression of activated CREB in the DRG changed over the course of the first 6 weeks after SCI, with the levels at 6 weeks being significantly greater than controls, though not in the DRG we examined here. Other studies demonstrate induction of CREB in injured/stressed neurons and also in neurons post-synaptic to stressed sensory neurons (e.g., Ji and Rupp, 1997; Bedogni et al., 2003; Choi et al., 2003; He et al., 2003; Zhu et al., 2004a), while others demonstrate CREB regulating multiple NTs (Bender et al., 2001), in at least one case by interacting with cytokines (Otten et al., 2000).

Unlike the results of the analysis of the gene promoter regions using the TransFac database, the set of miRNAs that emerged from the TargetScan analysis of gene 3′-UTRs were not shared across multiple genes. It should be noted that these analyses necessarily have certain differences that certainly impacted the results. Most notable is that TargetScan returns only those miRNA targets that have experimental confirmation. The data in Table 7 could therefore be considered a snapshot of the current experimental data regarding which miRNA species interact with those genes (Saba et al., 2008; Guidi et al., 2010; Natera-Naranjo et al., 2010; Rau et al., 2010; Smith et al., 2010; Benoit and Tenner, 2011; Farrell et al., 2011; Kawahara et al., 2011, 2012; Yu et al., 2011, 2012; Brandenburger et al., 2012; Hamada et al., 2012; Ryan et al., 2012; Wang et al., 2012). Thus, although our analysis revealed no common miRNA species that interacted with multiple genes (representing a possible common regulatory mechanism), this may yet be the case.

Because our data are derived from homogenized tissue, we cannot make any conclusions about the cellular basis of this apparent coordinated expression. That is, we cannot determine which aspect, if any, of this coordination is occurring within single cells, or if it is simply occurring within the same tissue but arises through expression of different combinations of genes across different cells. Considerations of this issue here are at best speculative as there are virtually no studies that can provide information directly relevant to the question. Relevant information would include (1) an indication of which types of cells were expressing the genes, or at least if they were neural, non-neural, or both, (2) an indication of whether or not any combination of the genes were expressed in any single cells, and both of these would (3) have to be sampled from DRG or SC 12 weeks after SCI. We are not aware of any studies fitting these criteria (Table 1). Although the relationship is not direct, we can nonetheless draw from a number of sources to make inferences about what may be happening.

(1) There is some evidence that at 6 weeks after SCI Trk receptors are expressed almost exclusively in DRG neurons, much as before the SCI (Qiao and Vizzard, 2002, 2005). However, it must be noted that there is a plethora of evidence of expression of NTs and Trks in non-neuronal cells (e.g., Funakoshi et al., 1993; Elkabes et al., 1998; Nemoto et al., 1998; Noga et al., 2002; Hess et al., 2007), although much of this is in the context of cancer (e.g., Tacconelli et al., 2005; Howe et al., 2011; Jin et al., 2011). Studies which identify the cell types expressing the NTs or Trks are necessary as it is possible that at least a portion of the tissue-level regulation could be due to invading cells. Certainly the complement of immune cells in the SC is affected by injury, even in segments spatially remote from the injury (e.g., Popovich et al., 1997). Immune cells invade the DRG after nerve injury (e.g., Nguyen et al., 2007; Vega-Avelaira et al., 2009; Kim and Moalem-Taylor, 2011), but there is no indication that this possibility has been examined in DRG at any time after SCI. However, evidence suggests that the immune cells and their functions throughout the body may be affected by SCI (e.g., Popovich et al., 2001), and some express Trk receptors and/or neurotrophins (e.g., Noga et al., 2002; Nassenstein et al., 2004; Tabakman et al., 2004).
(2) There is evidence that single neurons can express certain limited combinations of the genes examined here, though to our knowledge there has been no examination of all together that could distinguish each of the Trk receptors and neurotrophins (e.g., McMahon et al., 1994; Obata et al., 2004).
(3) There are certainly studies which examine the chronic post-SCI condition, but we could not identify any that could provide data relevant to these specific considerations (i.e., they examined other readouts).

Almost irrespective of the outcome of the above considerations, there is still another consideration that can be brought to bear. Although there are a number of papers describing co-expression of some of these genes in single cells where common genetic/molecular regulation could possibly be exerted, it is highly unlikely that all the coordinated expression is accounted for by single cells. Even in the feasible condition where expression is limited to neurons, and perhaps even to the same population of neurons that expressed these genes in the intact system (i.e., differences in expression would be based on volume regulation in any given cell and not on recruitment/de-recruitment of cell populations), what is the likelihood that this degree and scope of coordinated expression could occur across different cell types independently? It seems highly unlikely that each of the genes considered here would change in a single cell type independent of its regulation in any other cell type, and still give rise to this result. However, because there is little-to-no cellular expression data here or in the literature from which to extrapolate the identity of the cells expressing these genes (i.e., immunocytochemical or *in situ* hybridization assessment of SC or DRG 12 weeks post-SCI), we must acknowledge that this is indeed possible in principle. There is, however, virtually no reason to expect that individual cells would express all of the “coordinated” genes and thus have the mechanism of coordinated regulation exist fully inside of those given single cells. Therefore, at least some of the coordination must arise *across* cells which express one or more of the “coordinated” genes.

It is possible that coordinated regulation/expression may arise due to shared direct molecular mechanisms, but the literature and our bioinformatic analyses provide little evidence for a simple mechanism of this sort. There may yet be coordinated transcriptional regulation that is indeed shared across cell types, but may reside at a level above our analyses (i.e., shared factors may be directing the actions of separate factors that then individually act on the different genes). Alternatively, there may be a shared biological process(es) or response(es) that is being executed in the various different cells – a process that has similar outcomes in terms of gene regulation but arrives there through the actions of different specific molecular entities. For “simplicity,” let us consider that only the neurons of the DRG are involved. Even this cell population is not homogeneous in function, form, or sensitivity. Each of the Trk receptors is largely separately expressed. Given the dissimilarities of their regulatory sequences, they may each be directly regulated by distinct factors. However, conditions may arise that induce the non-homogeneous neuronal types, regardless of the specific Trk they express (and thus which specific factors will act on the DNA and/or mRNA), to coordinately regulate the expression of their Trk receptor. It is possible that the regulation of those specific factors may be under a control mechanism that is itself shared across the different neuron types. Our analysis would not detect this. As an example, consider cellular stress or injury. Numerous authors have reported on the regulation of Trks and neurotrophins in response to nerve injury, and the change in expression over time (e.g., Ernfors et al., 1993; Sebert and Shooter, 1993; Krekoski et al., 1996; Yamamoto et al., 1996; Bergman et al., 1999; Lee et al., 2001; Kuo et al., 2007), and many aspects of our data coincide with the reported regulation after nerve injury or neuronal stress. Intriguingly, there was another report of “coordinated regulation” associated with DRG neurons and glia in conditions of injury and/or stress (Cameron et al., 2003).

It is not clear if SCI induces any long-term injury or stress on DRG neurons. Certainly the central axons of some DRG neurons are damaged in the SCI, particularly those terminating in the affected cord, or with long axons ascending through the dorsal columns (Huang et al., 2006). However, the effect of injury to central axons differs from that of injury to peripheral axons (e.g., Stam et al., 2007), and the long-term effects on expression of neurotrophins and Trk receptors has not been examined. Injury to central axons is not the only possible source of stress to the sensory neurons, however. The inflammatory condition of the SC and continued spread of damage may induce injury or stress in the sensory neurons at times remote from the acute SCI, and at locations remote from the lesion (e.g., Popovich et al., 1997; Popovich, 2000; Bao et al., 2004, 2011; Fleming et al., 2006; Gris et al., 2008; Kwon et al., 2010; Lubieniecka et al., 2011; Ng et al., 2011; Stammers et al., 2012). There is a systemic inflammatory condition (Fleming et al., 2006; Gris et al., 2008; Bao et al., 2012) that has unknown effects on these neurons. Additionally, one must consider the effects of SCI on the peripheral tissues innervated by the sensory and motor neurons. The inflammation and altered activity/mobility/use state can impact these tissues (e.g., Edwards-Beckett and King, 1996; Lynch et al., 2000; Gris et al., 2008) with uncertain consequences for the innervating neurons. The increased expression of galanin, a neuropeptide induced in DRG neurons by stress/injury (Suarez et al., 2006), in the DRG innervating bladder and bowel (but not other DRG) after SCI (Zvarova et al., 2004) suggests that the histopathology secondary to SCI may stress the sensory neurons innervating those tissues. Tissue damage has been shown to induce stress/injury responses in sensory neurons (e.g., Ivanavicius et al., 2007; Hill et al., 2010; Thakur et al., 2012), and has been shown to affect regulation of multiple neurotrophins in the injured tissue (Vizzard, 2000).

### Regulation of neurotrophins and Trks after SCI: Enough assessment or not?

Although there are many reports examining the expression of neurotrophins and/or Trk receptors after SCI, there is relatively little overlap of the data (Table 1), and general principles have yet to be identified. That is not to say that the data disagree, but more that the studies have largely produced different data. Indeed, given the number of factors that influence gene regulation after SCI, much work is yet to be done. A matrix of variables demonstrated by our study and others to significantly impact the regulation of these genes suggests that over 1000 assessments would be required to provide relatively thorough coverage (Table 8). This matrix relates only to natural progression and does not include variables for the two sexes, different species and strains, and outcome measures (e.g., protein, mRNA, behavior, etc.). It would thus only be expanded when considering treatments, sex/species/strain-differences, and multiple outcome measures (some of which are mutually exclusive), each of which has been shown to influence the data (e.g., Popovich et al., 1997; Sroga et al., 2003; Kigerl et al., 2006; Beck et al., 2010).

**Table 8:** **Matrix of factors influencing outcomes in SCI research**.

Injury type/severity	Injury location	Region investigated	Post-SCI time
Hemisection – lateral	Cervical	Cervical	1–3 days
Hemisection – D/V	Brachial plexus	Brachial plexus	3–7 days
Transection	Thoracic	Thoracic	1–3 weeks
Contusion – mild	Lumbar	Lumbar	3–6 weeks
Contusion – moderate	Lumbar plexus	Lumbar plexus	6–12 weeks
Contusion – severe	Sacral	Sacral	12–24 weeks

This study examined only mRNA expression levels, which could change due to a limited set of non-mutually exclusive scenarios. Cells already expressing the specific transcripts could up- or down-regulate expression, or a different population of cells – resident or infiltrating – could begin expressing these transcripts *de novo*. Our data cannot speak to the relative contribution of these possibilities as they come from homogenized tissue. Although the literature provides some insight for the 6-week post-SCI data, this is not true for the 12-week data as neurotrophins and Trk receptors have not been examined at 12 weeks post-SCI (Table 1). Further, we are not aware of any work examining whether cells infiltrate the DRG after SCI.

## Conclusion

Despite the limitations of examining only mRNA expression, this study has established that different injury severities within the same model can result in different forms of regulation of these important genes in neural tissue. It has also demonstrated that expression of these genes in neural structures providing innervation to the hindlimb changes over a time course important for experiments examining activity-dependent plasticity and also for modeling the human condition. Thus, this study (1) offers insight for interpreting published data and for designing future studies; (2) serves as a reference for mechanistic studies that manipulate the neurotrophin-Trk signaling systems, (3) indicates that injury severity, post injury time, and tissue sampled all influence the assessments of gene regulation, (4) suggests that regulation of these genes continues to occur as late as 12 weeks post-SCI, and (5) suggests that some common factor or process may be influencing expression of these genes at later times after SCI.

## Conflict of Interest Statement

The authors declare that the research was conducted in the absence of any commercial or financial relationships that could be construed as a potential conflict of interest.
